# Mining Genes Involved in Insecticide Resistance of *Liposcelis bostrychophila* Badonnel by Transcriptome and Expression Profile Analysis

**DOI:** 10.1371/journal.pone.0079878

**Published:** 2013-11-20

**Authors:** Wei Dou, Guang-Mao Shen, Jin-Zhi Niu, Tian-Bo Ding, Dan-Dan Wei, Jin-Jun Wang

**Affiliations:** Key Laboratory of Entomology and Pest Control Engineering, College of Plant Protection, Southwest University, Chongqing, P. R. China; Natural Resources Canada, Canada

## Abstract

**Background:**

Recent studies indicate that infestations of psocids pose a new risk for global food security. Among the psocids species, *Liposcelis bostrychophila* Badonnel has gained recognition in importance because of its parthenogenic reproduction, rapid adaptation, and increased worldwide distribution. To date, the molecular data available for *L*. *bostrychophila* is largely limited to genes identified through homology. Also, no transcriptome data relevant to psocids infection is available.

**Methodology and Principal Findings:**

In this study, we generated *de novo* assembly of *L. bostrychophila* transcriptome performed through the short read sequencing technology (Illumina). In a single run, we obtained more than 51 million sequencing reads that were assembled into 60,012 unigenes (mean size = 711 bp) by Trinity. The transcriptome sequences from different developmental stages of *L. bostrychophila* including egg, nymph and adult were annotated with non-redundant (Nr) protein database, gene ontology (GO), cluster of orthologous groups of proteins (COG), and KEGG orthology (KO). The analysis revealed three major enzyme families involved in insecticide metabolism as differentially expressed in the *L. bostrychophila* transcriptome. A total of 49 P450-, 31 GST- and 21 CES-specific genes representing the three enzyme families were identified. Besides, 16 transcripts were identified to contain target site sequences of resistance genes. Furthermore, we profiled gene expression patterns upon insecticide (malathion and deltamethrin) exposure using the tag-based digital gene expression (DGE) method.

**Conclusion:**

The *L. bostrychophila* transcriptome and DGE data provide gene expression data that would further our understanding of molecular mechanisms in psocids. In particular, the findings of this investigation will facilitate identification of genes involved in insecticide resistance and designing of new compounds for control of psocids.

## Introduction

Psocoptera, also known as booklice or psocids, are frequently found in stored grains, often in extremely high numbers [1]. Although psocids were considered as nuisance pests rather than causative agents of losses to stored commodities [2], now there is a growing evidence of economic losses by psocid infestation of stored grains as well as psocid contamination of food and commodities [3]–[6]. Recent studies indicate that they pose a new risk for global food security and safety [7]. Moreover, development of psocids is also known in whole grains [7], [8]. Psocids are perhaps the most important category of emerging pests in stored grains and related commodities [7]–[9].

Routine fumigations of warehouses and storage facilities with methyl bromide have failed to control the pests [10]. In addition, the rapid development of resistance to chemical and physical treatments by psocids has also been reported [11]. Almost all currently registered grain protectants such as deltamethrin, permethrin and carbaryl fail to control psocids [12], thereby elevating their pest status enormously and putting them alongside the major beetle pests [13].

Among the psocids, *Liposcelis bostrychophila* Badonnel (Psocoptera: Liposcelididae) is important because of its parthenogenic reproduction, rapid adaptation, and increased worldwide distribution. To date, several studies have been performed on the ecology, toxicology, behavior, and physiology of *L. bostrychophila*, such as the population dynamics, insecticide evaluations, diet choice, resistance development and control [7], [9], [11], [14]–[16]. However, like in many important agricultural pest species, there is very limited amount of molecular biology study of *L. bostrychophila*. Prior to this study, there was only small number of putative gene sequences for *L. bostrychophila* available in the NCBI database. Furthermore, most of these were identified solely on the basis of homology to genes previously characterized in other related species. The lack of comprehensive molecular data severely constrains further molecular studies of *L. bostrychophila* on specific subjects including ecology, reproductive behavior, and population genetics [17]–[19]. For future research in areas such as the management of insecticide resistance, especially in species whose genomes have not been extensively characterized [17], it will be very helpful to have genome-scale molecular data to study mechanisms of insecticide resistance [19].

During the past several years, next-generation sequencing (NGS) technologies (including Solexa/Illumina, SOLID/ABI, 454/Roche, and Heliscope/Helicos) have been developed to produce massively parallel sequences in relatively short times and at considerable reductions in terms of cost and labor requirements [20]. NGS affords unprecedented high-throughput and low-cost sequencing for *de novo* whole-genome sequencing, resequencing of genomes to identify variations, *de novo* transcriptome and gene expression profiling, and detecting methylation patterns [21]–[23]. At present, the Solexa/Illumina sequencing technology dominates the NGS market for featuring high data accuracy and the broad range of applications [23].

The goals of the current study are (1) to assemble as many as possible *L. bostrychophila* transcripts from Illumina sequencing reads, (2) to analyze the expression levels of genes in the genome of this species through digital gene expression (DGE) profiling and (3) to find a broad range of genes in this species putatively involved in insecticide resistance. To achieve these goals, *de novo* transcriptome assembly and DGE profiling analysis were performed.

## Results

### *De novo* Sequence Assembly of the Transcriptome

**Figure 1:**
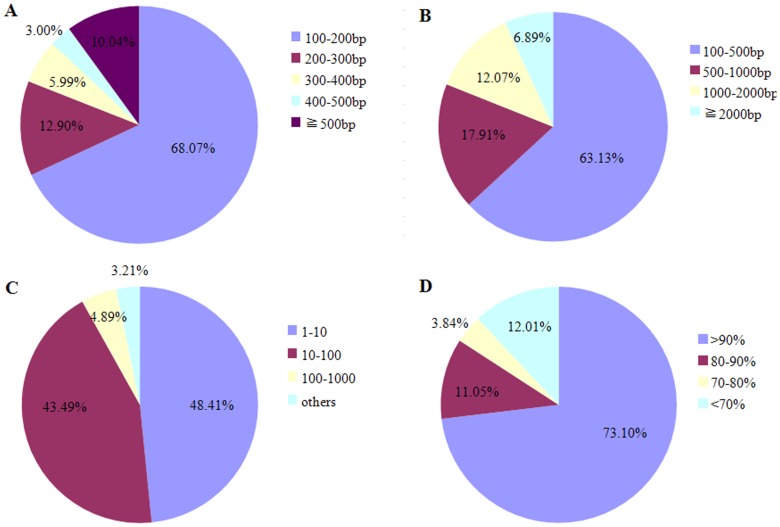
Overview of transcriptome sequence assembly.

To obtain a comprehensive transcriptome of *L. bostrychophila*, a cDNA library was constructed using RNA isolated from eggs, nymphs and adults and pooled with equal ratios. A total of 51,336,216 clean reads of 90 bp long (consisting of 4,620,259,440 bp) were produced after removal of the dirty reads from the raw reads (Table 1). This represented more than 4.6 Gbps of genomic sequences of the psocids generated by Illumina HiSeq™ 2000 sequencing. The sequencing quality of the clean reads was evaluated based on the base-calling quality scores of Illumina’s base-caller Bustard. More than 97% of the clean reads had quality scores higher than the Q_20_ level (an error probability of 0.01). These high-quality clean reads were assembled *de novo* using the Trinity program, resulting in 172,663 contigs longer than 100 bp with a mean length of 278 bp. Although the majority of the contigs were between 100 and 200 bp (68.07% of total), 17,331 (10.04%) were longer than 500 bp (Fig. 1A). Finally, the contigs were further assembled into 60,012 unigenes, which included 13,534 distinct clusters and 46,478 distinct singletons. The mean length of unigenes was 711 bp, with 11,379 unigenes longer than 1,000 bp and 4,137 unigenes longer than 2,000 bp (Fig. 1B). To evaluate the quality of the dataset, the sequencing depth and coverage were also calculated. A majority of unigenes (91.90%) exhibited depths ranging between 1 and 100 (Fig. 1C), while 84.15% of unigenes possessed coverage values over 80% (Fig. 1D).

**Table 1:** Overview of *Liposcelis bostrychophila* cDNA library assembly and annotation.

Sequencing	
Total number of clean reads	51,336,216
Total number of clean nucleotides	4,620,259,440
Assembly	
Number of contigs	172,663
Number of unigenes	60,012
Annotation	
Unigenes annotations against Nr	24,686
Unigenes annotations against Swiss-Prot	19,916
Unigenes annotations against KEGG	17,985
Unigenes annotations against COG	9,690
Unigenes annotations against GO	13,997

Nr, Nonredundant database; KEGG, Kyoto Encyclopedia of Genes and Genomes database; COG, Clusters of Orthologous Groups database; GO, Gene Ontology database.

### Annotation of Predicted Proteins

**Figure 2:**
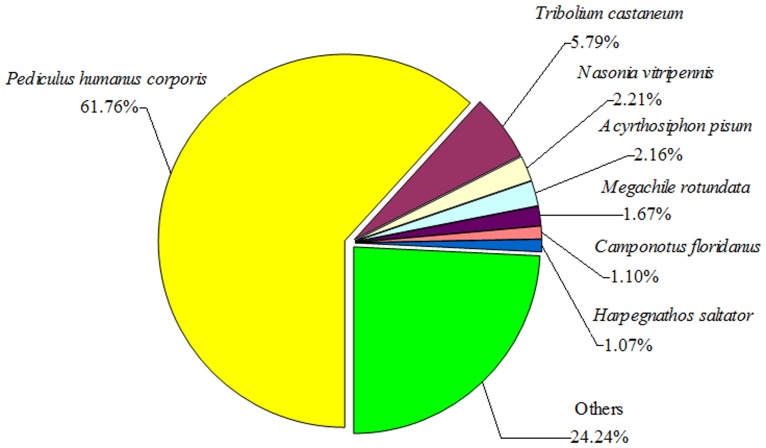
Species distribution of homology search of unigenes against the Nr database. The species distribution is shown as the percentage of the total homologous sequences in the NCBI Nr protein database with an E-value<10^−5^.

To annotate these unigenes, distinct sequences were first searched by BLASTx against the NCBI non-redundant protein database (Nr) with a cut-off *E*-value of 10^−5^. In total, 24,686 unigenes (41.14% of all distinct sequences) matched known genes that encoded functional proteins (Table 1). The species distribution of the top BLAST hits for each unique sequence is shown in Fig. 2. The unambiguous unigenes from *L. bostrychophila* revealed that the greatest number of matches (61.76%) was with sequences from the Anoplura species *Pediculus humanus corporis* followed by the sequences from *Tribolium castaneum* (5.79%), *Nasonia vitripennis* (2.21%), *Acyrthosiphon pisum* (2.16%), *Megachile rotundata* (1.67%), *Camponotus floridanus* (1.10%), and *Harpegnathos saltator* (1.07%).

### Unigene Functional Annotation by GO, COG, and KEGG

**Figure 3:**
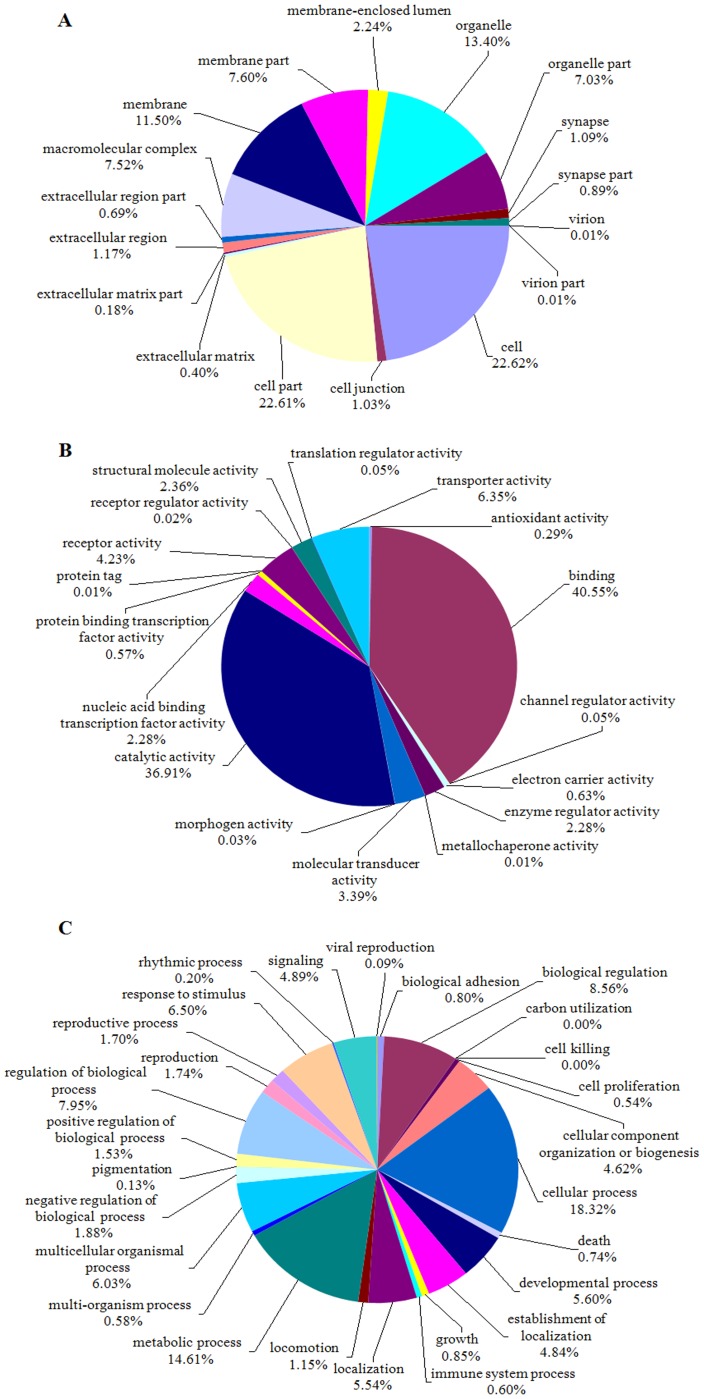
Gene ontology annotation and classification of the *Liposcelis bostrychophila* transcriptome.

Gene Ontology (GO) is an international standardized gene functional classification system. In total 13,997 unigenes were annotated as 89,865 GO terms. However, some of these unigenes participated in multiple GO terms. They were divided into three categories and 61 sub-categories (Fig. 3): biological process (27 sub-categories), cellular component (17 sub-categories) and molecular function (17 sub-categories). However, the majority of the GO terms represented biological process (44,023; 48.99% of the total). This was followed by GO terms of cellular component (27,263; 30.34%) and molecular function (18,579; 20.67%). The three major sub-categories were cellular process (8,065 GO terms) in the biological processes category and binding (7,534 GO terms) and catalytic activity (6,857 GO terms) in the molecular function. The smallest groups were cell killing in the biological process category and protein tag in the molecular function, both with only one GO term. When all 60,012 unigenes were aligned against the Swiss-Prot database using BLASTx, a total of 19,916 unigenes yielded a significant hit to one or more proteins.

**Figure 4:**
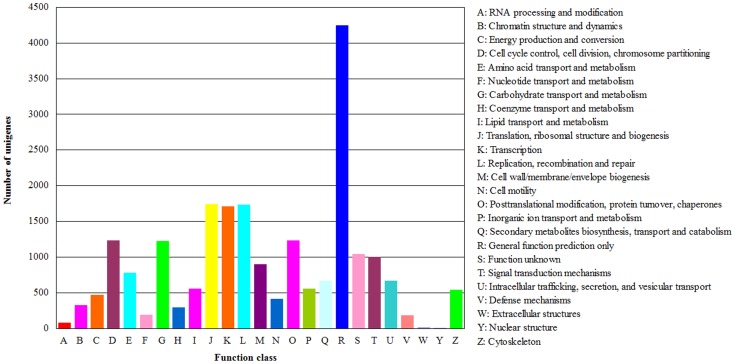
Clusters of orthologous groups (COG) predicted from the unigenes.

All assembled unigenes were aligned to the COG database for functional prediction and classification. A total of 9,690 COG annotations were identified for 21,875 annotated unigenes, which were classified into 25 molecular families (Fig. 4). Thus, some of these unigenes were associated with multiple COG annotations. Among these functional classes, the ‘‘general function prediction only” cluster constituted the largest group (4,251; 19.43%), followed by ‘‘translation, ribosomal structure and biogenesis’’ (1,747; 7.99%), and ‘‘replication, recombination and repair’’ (1,732; 7.92%). The two smallest groups were ‘‘extracellular structures’’ (19; 0.09%) and ‘‘nuclear structure’’ (8; 0.04%).

To identify the metabolic pathways populated by these unigenes, all assembled unigenes were mapped to the Kyoto Encyclopedia of Genes and Genomes (KEGG) pathways (Table S1). A total of 17,985 unigenes were annotated to 253 KEGG pathways. The pathways with the most unigenes were ‘‘metabolic pathways’’ (2,254; 12.53%), followed by “Regulation of actin cytoskeleton” (701; 3.90%) and “Focal adhesion” (668; 3.71%); ‘‘Butirosin and neomycin biosynthesis’’ (3; 0.02%) and ‘‘Asthma’’ (2; 0.01%) pathways were populated by the fewest number of unigenes. These annotations laid the groundwork for further research of metabolic pathways, functions, and biological role of *L. bostrychophila* genes.

### Transcripts Encoding the Insecticide Detoxification and Target Enzymes

The unigenes relating to insecticide resistance were identified. They generally encode detoxifying enzymes. Generally, the cytochrome P450s (P450s, EC 1.14.14.1), the carboxylesterases (CESs, EC 3.1.1.1), and the glutathione *S*- transferases (GSTs, EC 2.5.1.18) are the three primary enzymes involved in the detoxification of insecticides.

**Figure 5:**
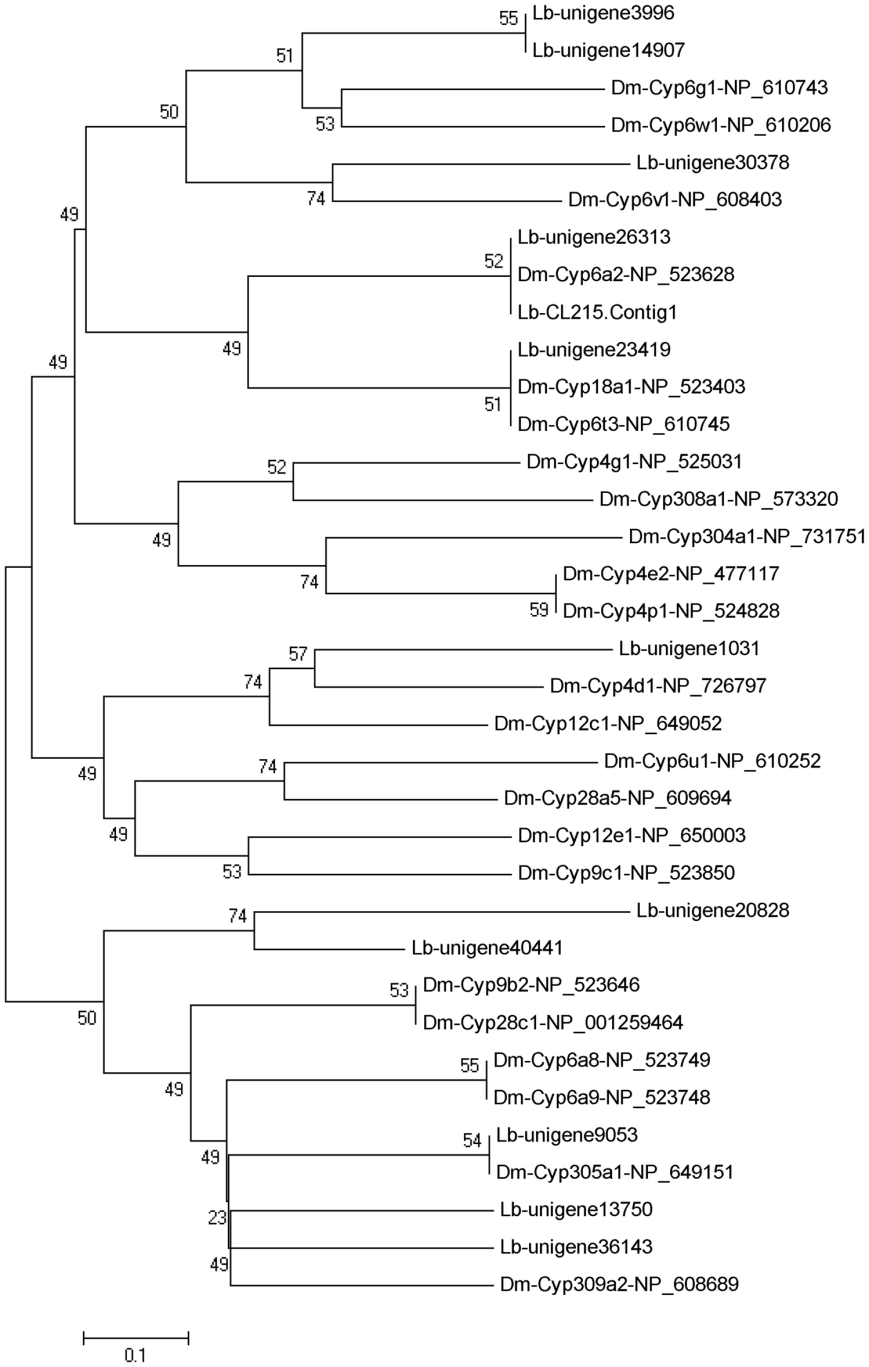
Phylogenetic tree of cytochrome P450s from *Liposcelis bostrychophila* (Lb) and *Drosophila melanogaster* (Dm). The tree was constructed from the multiple alignments using MEGA 5.0 software and generated with 1,000 bootstrap trials using the neighbor-joining method. The numbers indicate the bootstrap confidence values obtained for each node after 1,000 repetitions.

A total of 80 P450-related unigenes were identified from the Nr annotation of the *L. bostrychophila* transcriptome. After manually removing unigenes with lengths <300 bp or short open reading frames (ORFs), the remaining 49 P450 unigenes were assigned to the appropriate CYP (cytochrome P450) clades based on their closest BLAST hits in the NCBI Nr database. The psocid P450s could be divided into three major families, including 19 to CYP4, 18 to CYP6, and the remaining 12 P450s belonging to other families. Among the 49 P450-specific genes, 12 full length genes were identified. Their average length was 769 bp. Based on the phylogenetic analysis with P450 genes from *Drosophila melanogaster*, they belonged to CYP4, CYP6, CYP9, CYP12, CYP18A, CYP28, and CYP304-CYP309 families and subfamilies (Fig. 5).

**Figure 6:**
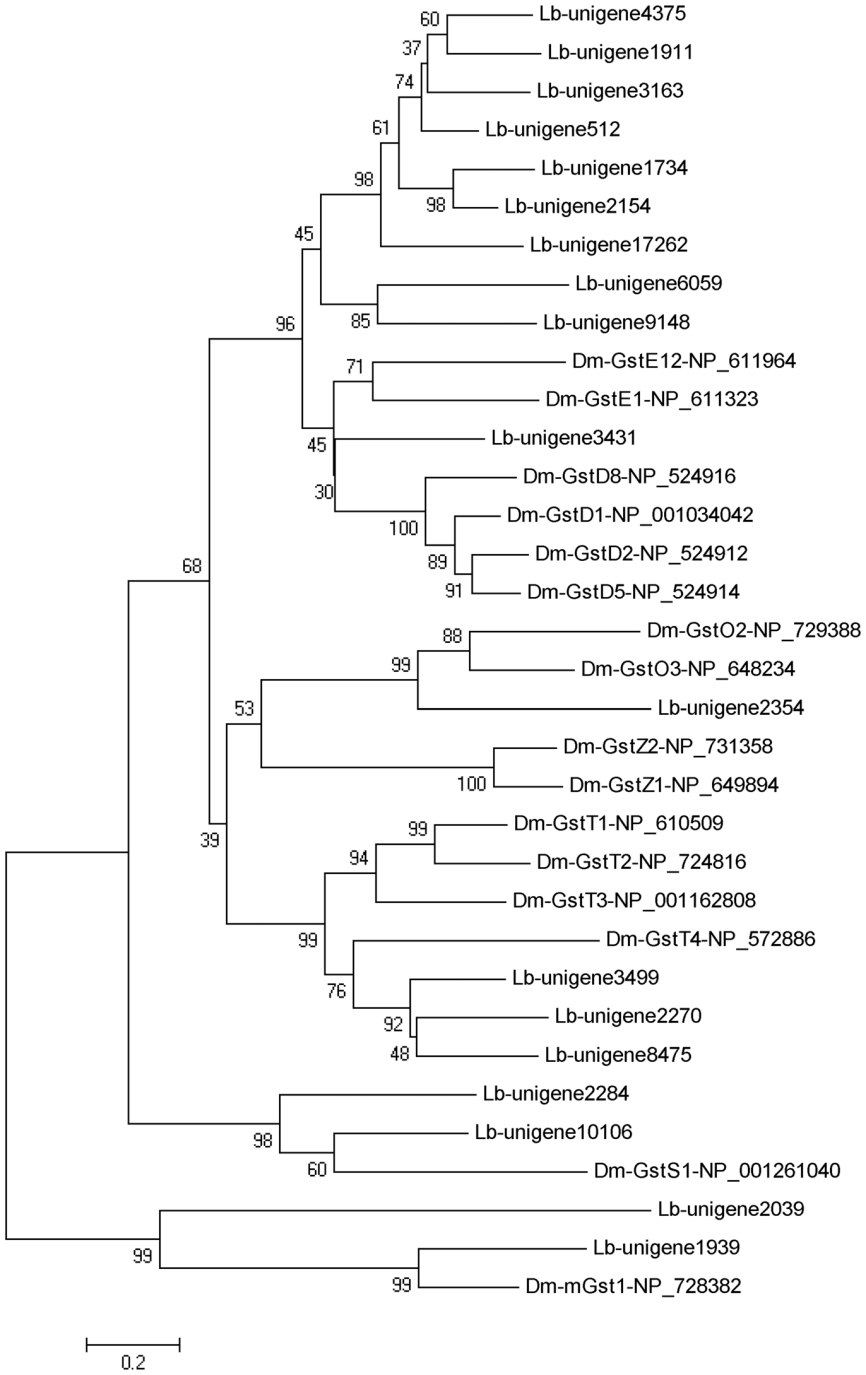
Phylogenetic tree of glutathione S-transferases from *Liposcelis bostrychophila* (Lb) and *Drosophila melanogaster* (Dm). The tree was constructed from the multiple alignments using MEGA 5.0 software and generated with 1,000 bootstrap trials using the neighbor-joining method. The numbers indicate the bootstrap confidence values obtained for each node after 1,000 repetitions.

We also identified 36 GSTs unigenes from Nr annotation of the *L. bostrychophila* transcriptome, 5 of which were removed for short length transcripts. Among the 31 GSTs-specific genes, 18 full length genes with an average length of 954 bp were identified by comparing with GSTs genes from *D. melanogaster*. Phylogenetic analysis showed that these genes were assigned to Epsilon, Delta, Omega, Theta, Sigma, and Microsomal class of GSTs (Fig. 6).

**Figure 7:**
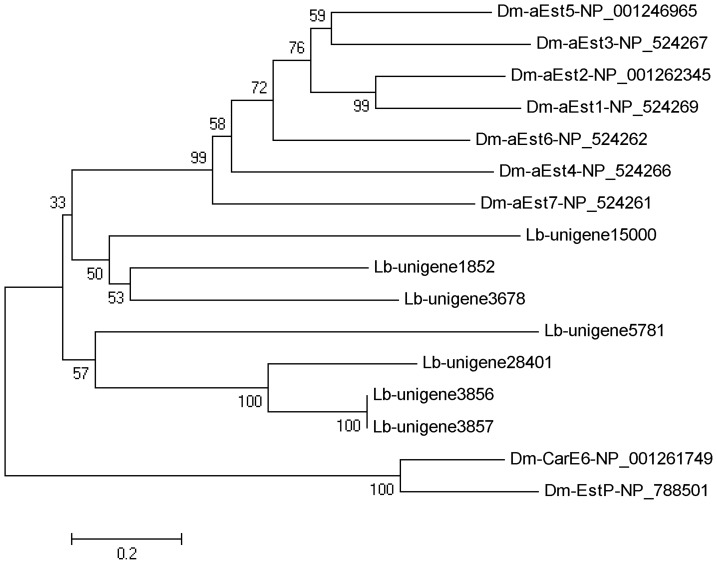
Phylogenetic tree of carboxylesterases from *Liposcelis bostrychophila* (Lb) and *Drosophila melanogaster* (Dm). The tree was constructed from the multiple alignments using MEGA 5.0 software and generated with 1,000 bootstrap trials using the neighbor-joining method. The numbers indicate the bootstrap confidence values obtained for each node after 1,000 repetitions.

A total of 76 unigenes related to CESs were identified. Of these, 21 were manually removed because they were either esterases or were short sequences or allelic variants. Among the 55 CES-specific genes, 7 full length genes were identified with an average length of 1,583 bp. Phylogenetic comparison with genes from *D. melanogaster* indicated that these sequences had homology with a-esterases (Fig. 7).

Unigenes encoding insecticide target proteins were also identified from the Nr annotation database of the *L. bostrychophila* transcriptome. They included acetylcholinesterase (AChE), voltage gated sodium channel (VGSC), nicotinic acetylcholine receptor (nAChR), and gamma-aminobutyric acid (GABA) receptor. For VGSC, the longest unigene (unigene 16,144) was chosen as representatives of the target proteins (Table 2). In addition, ten nAChR unigenes (nine alpha subunits and one beta subunit) and four GABA receptor unigenes (one each of GluCl, GABA-A, GABA-B and GABARR3) were also identified.

**Table 2:** Unigenes associated with insecticides target sites in *Liposcelis bostrychophila.*

Insecticide class/target site	Gene name	Gene ID	Length (bp)
Pyrethroids, pyrethrins/voltage-gated sodium channel	VGSC	16144	1172
Neonicotinoids, spinosad/nicotinic acetylcholine Receptor	nAChR alpha 1 subunit	19071	931
	nAChR alpha 2 subunit	22583	232
	nAChR alpha 3 subunit	16982	1058
	nAChR alpha 4 subunit	20590	1142
	nAChR alpha 6 subunit	31313	364
	nAChR alpha 7 subunit	8103	992
	nAChR alpha 8 subunit	16340	745
	nAChR alpha 9 subunit	11736	988
	nAChR alpha 10 subunit	5603	934
	nAChR beta 1 subunit	5673	928
Cyclodiene, fipronil/gamma-aminobutyric acid(GABA)-regulated chloride channel	GluCl	15532	1565
	GABA-B	17881	552
	GABA-A	1992	562
	GABARR3	9664	827
Organophosphate, carbamates/acetylcholinesterase	AChE	8358	2052

### Digital Gene Expression (DGE) Library Sequencing

**Figure 8:**
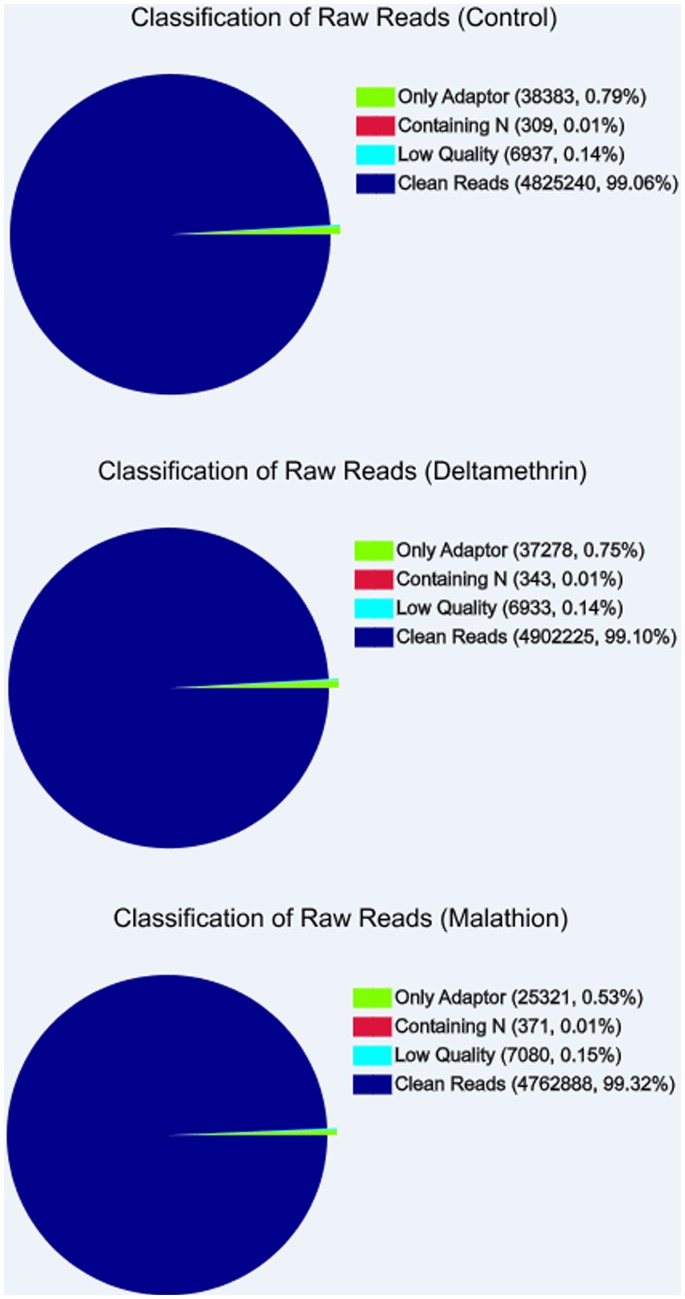
Evaluation of the sequence quality of the digital gene expression libraries.

To measure the absolute mRNA expression levels of *L. bostrychophila* under different insecticides exposures, gene expression variations were analyzed by the DGE approach. Three DGE libraries of *L. bostrychophila* were sequenced: control, deltamethrin-exposed, and malathion-exposed. We generated more than five million raw reads in each library. The total numbers of clean reads were 4.8 million, 4.9 million, and 4.8 million, respectively, after removing the low-quality reads. The clean reads comprised >99% of the raw reads in each library indicating the high quality of the sequencing (Fig. 8). For mRNA expression, heterogeneity and redundancy are two significant characteristics. While the majority of mRNA is expressed at low levels, a small proportion of mRNA is highly expressed. Therefore, the distribution of unique reads was used to evaluate the normality of our RNA-Seq data. The percentage of the clean reads that could be mapped to unigenes was 70.18%, 70.20%, and 69.39%, respectively. More than 4% of genes had 90–100% coverage in each library, while 6% of the genes had 0–10% unigenes coverage. More than 34% of the genes in each library had coverage higher than 50%.

### Gene Expression Profiles After Deltamethrin and Malathion Exposure

**Figure 9:**
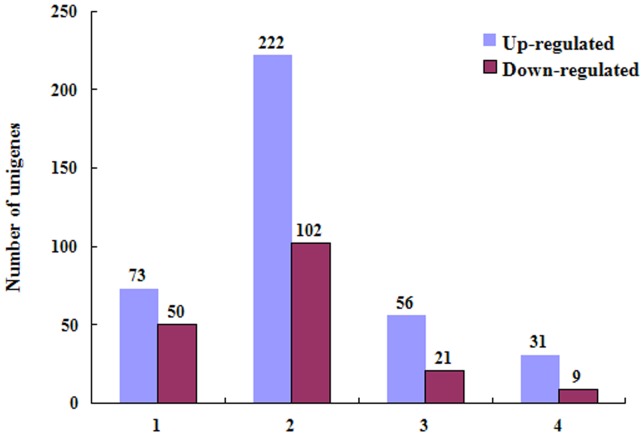
Summary of differently expressed unigenes in each pairwise comparison. 1: comparison between deltamethrin-exposed and control psocids; 2: comparison between malathion-exposed and control psocids; 3: differentially expressed unigenes which are common in the two previous comparisons; 4: common differentially expressed unigenes annotated by the Nr database.

To identify differentially expressed genes after exposure to pesticide, differences in gene expression were analyzed by pairwise comparisons of control and deltamethrin-exposed psocids, as well as control and malathion-exposed psocids. We found that 73 and 50 unigenes were significantly up- or down-regulated, respectively, in the control vs deltamethrin-exposed comparison (Fig. 9). When comparing the control and malathion-exposed treatments, 222 and 102 unigenes were significantly up- or down-regulated, respectively. Among the differentially expressed unigenes in the two comparison groups, 56 were up-regulated and 21 were down-regulated in both. We were able to annotate 31 of the 56 commonly up-regulated unigenes and 9 of 21 commonly down-regulated unigenes using the Nr database while the others encoded proteins with unknown functions (Fig. 9). Of the 40 annotated genes, six encoded ATP\GTP related protein, three encoded Trypsin and three encoded Cytochrome P450. These were the largest three groups of unigenes significantly over transcribed by insecticide exposure. Thirty three of the 40 unigenes with predicted functions could be annotated using the KEGG database, with 33 pathways involved (Table 3).

**Table 3:** Unigenes which are significantly differentially expressed can be annotated by Nr function annotation in both deltamethrin and malathion exposure.

Gene ID	Nr annotation	Expression changed	KEGGannotation	Pathway involved	log_2_ratio		*P*-value	
					D	M	D	M
128	GTP-binding nuear protein	Up	K07936	Ribosome biogenesis in eukaryotes, RNA transport	1	2	2E-22	8E-59
648	GTP-binding nuear protein	Up	K07936		1	2	1E-09	9E-14
1664	ATP-dependent RNA helicase	Up	K13184		1	2	4E-07	1E-23
1697	GTPase activating protein	Up	K04352	MAPK signaling pathway, Axon guidance	1	2	2E-42	1E-101
1715	ATP-dependent RNA helicase	Up	K13184, K15434		2	3	3E-51	7E-106
5387	GTP	Up	K01596	Citrate cycle, Adipocytokine, PPAR and insulin signalingpathway, Glycolysis	2	4	2E-33	0
2125	Trypsin	Up	K01312	Neuroactive ligand-receptor interaction, Pancreatic secretion, Protein digestion and absorption	3	2	4E-268	6E-232
2528	Trypsin	Up	K01312		2	2	9E-40	1E-51
3294	Trypsin	Up	K01312,		1	2	3E-16	2E-70
			K01324, K08667					
69	Chitinase	Up	K01183	Amino sugar and nucleotide sugar metabolism	2	1	9E-109	3E-28
352	Hypothetical protein	Up			3	3	9E-08	1E-07
518	Endochitinase	Up	K13909	Salivary secretion	3	3	3E-07	2E-06
1421	Fatty acyl-CoA reductase	Up	K13356	Peroxisome	1	1	2E-08	2E-12
1782	Apolipoprotein	Up	K15463, K14462	Fat and vitamin digestion and absorption	2	2	0	0
1902	PAX-interacting protein	Up	K14972		1	3	3E-40	2E-184
1917	Vicilin	Up	K13172		3	2	1E-62	1E-37
1969	Zinc transporter	Up	K14712		2	1	3E-260	9E-67
1991	Zinc finger protein	Up	K11308, K11432		1	2	9E-110	0
2189	Trehalase	Up	K01194	Starch and sucrose metabolism	1	1	3E-07	1E-09
2777	Dimethylaniline monooxygenase	Up	K00485		1	3	3E-31	1E-248
2862	Keratin	Up	K07604	Pathogenic *Escherichia coli* infection	8	9	7E-124	2E-254
3487	Keratin	Up	K07604		15	15	3E-09	4E-12
2896	RlpA family protein	Up			1	2	4E-15	9E-75
3499	Glycoprotein	Up	K10955	Vibrio cholerae infection, Amoebiasis	1	1	4E-18	1E-44
4005	Hypothetical protein	Up	K05746	Regulation of actin cytoskeleton	2	1	1E-23	2E-13
4829	Serine protease	Up	K01312	Pancreatic secretion, Protein digestion and absorption	2	3	4E-26	8E-102
6089	Serine protein	Up	K13171, K13172	RNA transport, mRNA surveillance pathway	2	2	7E-09	5E-09
7224	Decarboxylase	Up	K01594, K01580	Alanine, aspartate, glutamate, taurine and hypotaurine metabolism, Metabolic pathways,	1	1	4E-09	3E-06
12285	Hypothetical protein	Up		RNA degradation, Endocytosis, Morphine addiction, Focal adhesion, ECM-receptor interaction	2	2	6E-06	4E-08
32368	Cytochrome P450	Up	K15001	Amino sugar and nucleotide sugar metabolism	5	5	1E-07	2E-08
37036	Cytochrome P450	Up	K07427	Ribosome biogenesis, RNA transport	15	14	1E-09	1E-06
3024	Cytochrome P450	Down	K15001		−1	−2	3E-26	3E-68
1804	Thaumatin	Down			−1	−1	2E-85	5E-40
2235	Checkpoint protein	Down			−17	−2	1E-45	5E-20
2311	Carboxylesterase	Down	K01063, K03927	Insect hormone biosynthesis, Drug metabolism	−2	−2	2E-20	8E-18
2830	Vitellogenin	Down			−1	−2	4E-08	4E-19
3407	Entactin	Down	K06826		−1	−1	1E-19	3E-26
3514	Fumarylacetoacetase	Down	K01555	Metabolic pathways, Tyrosine metabolism	−1	−1	3E-08	8E-11
3715	Phosphorylase	Down	K03783	Metabolic pathways, Purine, pyrimidine, nicotinate and nicotinamide metabolism	−1	−1	4E-14	6E-13
23860	RlpA family protein	Down			−2	−3	1E-10	1E-13

KEGG, Kyoto Encyclopedia of Genes and Genomes database. The parameter of log_2_Ratio represents folds of different expression. D and M represent Deltamethrin/control and Malathion/control, respectively.

### Validation of Gene Expression by Quantitative Real-time PCR

**Figure 10:**
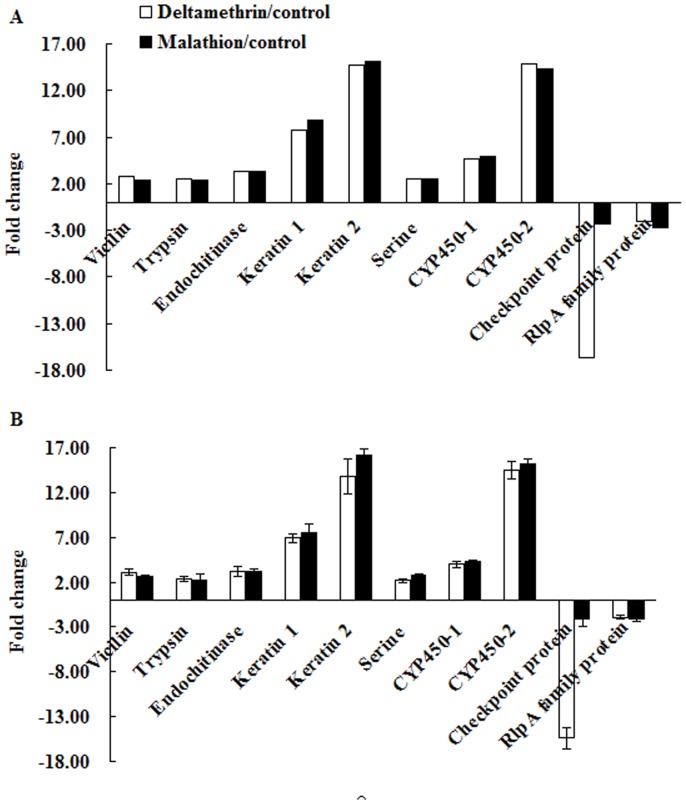
A validation of gene expression between control and insecticide treatments for 10 differentially expressed unigenes. A: Gene expression analysis from DGE data. The fold changes in gene expression were calculated as the log_2_ ratio of deltamethrin or malathion to control. B: qRT-PCR analysis of gene expression.

Quantitative real-time reverse transcription PCR (qRT-PCR) was conducted to validate the expression levels of genes from DGE sequencing. Of the 40 annotated genes both up- or down-regulated in the two comparison groups above, 10 genes (*vicilin*, *trypsin*, *endochitinase*, *keratin 1* and *2*, *serine*, *CYP450 1* and *2*, *checkpoint protein*, and *rlpA family protein*) were chosen randomly to perform qRT-PCR (Fig. 10A and Table S2). The results showed that both *checkpoint protein* and *rlpA family protein* had much lower expression levels in deltamethrin/malathion treatments compared to control, while other 8 genes were both over expressed significantly in insecticides treatments. All of these genes were amplified by qRT-PCR, and the results were consistent with those acquired by DGE profiling (Fig. 10B).

## Discussion

Psocoptera is a relatively small order of insects with approximately 4,400 species worldwide [24]. Managing psocid infestations in stored commodities has become increasingly difficult due to development of resistance to the most commonly used fumigants (such as phosphine, methyl bromide and ethylene dibromide) [10] and controlled atmospheres [11]. *L. bostrychophila*, a member of the order Psocoptera, was first described from the bark of cotton trees in Africa but has subsequently been found in most parts of the world, often associated with humans, their food stores and habitations [3]. This insect reproduces rapidly by obligatory thelytokous parthenogenesis and infestations of *L. bostrychophila* are particularly invasive [6], [14]. The species is regarded as one of the most difficult species to control among major Liposcelididae pest species and commonly found in various processed and unprocessed dry foods in households, granaries, and warehouses [7], [18]. The genome of *L. bostrychophila* determines its characteristics. Therefore, an exhaustive understanding of molecular mechanisms of *L. bostrychophila* behavior and activities may allow us to control the stored pest with more sustainable and environmentally friendly approaches.

Development of resistance to certain classes of insecticides such as phosphine, organophosphates and pyrethroids has been reported in *L. bostrychophila* and other psocids [7], [12], [15]. For many of these cases, the main mechanisms of insecticide resistance involve either enhancement of detoxification processes and/or modification of target sites. With respect to metabolic and detoxification processes, three groups of enzymes namely P450s, CESs, and GSTs are known to have role in developing insecticide resistance [19], [25]. The conservation of proteins in these families across species is universally not high, and the attempt to identify homologs of these genes through DNA sequence similarity is usually more challenging due to the substantial differences in codon usage patterns [19].

The genomic and transcriptome data obtained from the current study provide useful information to facilitate further investigation on resistance mechanisms and management strategy of the pest. The comparative genome analyses conducted in this study suggested both similarities and differences in the numbers of the three main detoxification enzymes involved in developing insecticide resistance. For example, the number of GSTs in *L. bostrychophila* was 31. This number is well within the range of 10 to 38 genes found for *Apis mellifera* and *D. melanogaster* [26], [27]. The number of P450 genes in *L. bostrychophila* (49 genes) is also within the range of 37–180 reported for other insect species. For example, the smallest number of P450 genes was reported in the human body louse *P. humanus*, while the largest is seen in the mosquito *Culex pipiens* [27], [28]. Similarly, the number of CES transcripts was 55 in *L. bostrychophila*. However, this number is higher than the known range of 24 to 51 genes in the *A. mellifera* and *Anopheles gambiae* genomes [27]. The BLASTx annotation of *L. bostrychophila* transcriptome sequences revealed the highest similarity (61.76%) with *P. humanus corporis* of the order Phthiraptera, far higher than the identity match (5.79%) with *T. castaneum*, a representative of the order Coleoptera and other species. The result is unsurprising considering the fact that psocids in the genus *Liposcelis* are closely related to parasitic lice of mammals (order Phthiraptera) [6].

A total of eight classes of GSTs are known [25], among which delta and epsilon GSTs are unique to insects and appear to play important roles in xenobiotic detoxification and insecticide resistance [25], [29]. In the *L. bostrychophila* transcriptome, 13 (almost 42%) of the GSTs identified from the transcriptome data belong to the Delta and Epsilon classes. Hence it is likely that these may have similar roles in xenobiotic detoxification in *L. bostrychophila*. Among the other classes of GSTs, for example the omega class, the number of GST genes found in *L. bostrychophila* (1) was consistent with those from *An. gambiae*, *A. mellifera*, and *P. humanus* [27], [30], [31]. For the sigma and theta class of GSTs, the number of genes in *L. bostrychophila* (5, 9) was larger than those of the *A. mellifera* (4, 1), *An. gambiae* (1, 2) and *D. melanogaster* (1, 4) [27], [30]. However, the theta class of genes was originally neglected due to the lack of affinity to GSH matrices and lack of activity with CDNB for which they are often referred to as the universal GST substrate [32]. Finally, the microsomal class of GSTs is designated as a group of membrane associated proteins involved in eicosanoid and glutathione metabolism (MAPEG) [32]. Identification of two genes in this class in *L. bostrychophila* is also consistent with that found in *A. mellifera* (2) and *P. humanus* (2) [30], [31]. However, this class has not been implicated in the metabolism of insecticides other than protective role in oxidative stress [27], [29].

The P450 genes represent a large and highly diverse gene family in different species. Some P450s (e.g. *CYP4E2* and *CYP4G8* in CYP4, *CYP6A1* and *CYP6A2* in CYP6) are of particular interest because of their crucial functions in detoxification of xenobiotics such as insecticides and plant toxins [33], [34]. For example, the *An. gambiae* contains 106 P450 genes [31] whereas *D. melanogaster* has only 85 P450 genes [34]. In our study, a total of 49 P450 type genes were identified in *L. bostrychophila*. This number is close to the numbers of P450 genes in *A. mellifera* (46) and *P. humanus* (37), while far less than that of *D. melanogaster* and *An. gambiae*. The *L. bostrychophila* P450 genes identified here are classified into 11 families. The largest family is CYP4 followed by the CYP6 family. In insects, the P450 genes known to be involved in insecticide resistance belong to CYP 4, 6, 9 and 12 families [35]–[37]. A total of 37 genes identified in *L. bostrychophila* belong to these families. Thus, it is likely that these P450 genes have roles in developing insecticide resistance in *L. bostrychophila*.

The CESs can be divided into 13 clades including the acetylcholinesterase (AChE) enzyme. These clades are classified into three classes consisting of enzymes with dietary/detoxification, pheromone/hormone processing, and neuro/developmental functions. In *L. bostrychophila*, a total of 55 putative CES genes are found. This number is close to 51 identified in *An. gambiae* but higher than that of *A. mellifera* and *P. humanus*, possessing only 17 genes. To our best knowledge, the *P. humanus* genome encodes the smallest number of detoxification enzymes observed in insect which be studied to date [31].

We identified a total of four types of target-site sequences related to different classes of insecticides by the BLASTx searches for genes in the NCBI database. Modification of AChE can lead to insensitivity to a variety of organophosphate and carbamate based compounds [38]. In our study, one unigene of 2,052 bps in length was identified as containing the *ace* gene, and it showed 99% similarity with a gene cloned and characterized from the same *L. bostrychophila* line [38]. The cases of knockdown resistance to pyrethroids, DDT resistance have been documented through reduced sodium channel sensitivity [39]. For VGSC, the longest unigenes of 1,172 bps (unigenes 16144) was chosen as representatives of the target proteins, though much less than the expected length about 6.5 Kbps in other insects [40]. The nicotinic acetylcholine receptors (nAChRs) represent a diverse family of cys-loop ligand-gated ion channels. Ten to twelve nAChR type receptor gene families have been reported in insects [41]. In *L. bostrychophila*, 9 alpha and 1 beta subunits have also been identified as nAChRs. Of these, two have sequences of more than 1,000 bps in length. In contrast, 12 nAChRs gene families are identified in both *Bombyx mori* [42] and *T. castaneum* [43] while *An. gambiae* and *Bactrocera dorsalis* have 10 such gene families [19], [44]. Finally, the gamma-aminobutyric acid (GABA) receptors also belong to the super family of cys-loop neurotransmitter receptors. Insect GABA receptors are divided into three classes, among which the modification of the GABA-regulated chloride channel can lead to resistance to dieldrin and fipronil [45]. In *L. bostrychophila*, one unigene of 1,565 bps (unigene 15532) were identified as GluCl indicating these classes of genes are also present in the psocid species.

Studying the temporal transcriptome responses of insects to xenobiotics could lead to the discovery of novel molecular mechanisms contributing to insecticide detoxification and tolerance [46]. To the best of our knowledge, our work represents the first analysis of the *L. bostrychophila* DGE profiles that greatly complement and enrich the psocid gene expression data. This will also facilitate the discovery of novel genes, gene functions and insecticidal targets of *L. bostrychophila*. Three DGE libraries of *L. bostrychophila* were generated after short-term exposure to sub-lethal concentrations of deltamethrin and malathion, which are commonly used as grain protectants to control psocids in stored products. Although deltamethrin and malathion belong to different chemical groups, 56 were up-regulated and 21 were down-regulated by these treatments compared to the control psocids. Thirty one of the 56 commonly up-regulated unigenes and nine from twenty one commonly down-regulated unigenes could be annotated, respectively. The other genes encoded proteins with unknown functions.

In insect cells, response to environmental stress trigger expression of various proteins including heat shock proteins [47], metallothioneins [48] or p-glycoprotein synthesis [49]. Although differentiating between xenobiotic-specific and general stress responses is difficult, our data highlighted that protein families such as ATP/GTP related proteins and glycoprotein family (the largest groups of 40 annotated unigenes) are significantly over transcribed by protectant exposure. Moreover, some genes encoding enzymes involved in the production of energy and cellular catabolism such as trypsin, trehalase, and serine were also over transcribed in psocids in response to xenobiotics. Other unigenes encoding enzymes such as chitinase and endochitinase were also found up-regulated in psocids when exposed to organic compounds. The rates of insecticide penetration have been found to be affected by thickened cuticles as well as by other structural components of cuticles including surface hydrocarbons [50]. Thus, a decrease in rates of penetration across the cuticle slows down transport of insecticides to internal organs and allows more effective metabolically mediated detoxification. The up-regulation of cuticular related proteins in *L. bostrychophila* indicates that cuticular protein thickening may have a role in reduced penetration of insecticide.

In conclusion, the present study represents a functional genomic analysis of *L. bostrychophila* to provide essential information to facilitate identification of genes involved in insecticide resistance, and may assist in designing new compounds or other strategies for the control of stored products pests.

## Materials and Methods

### Ethics Statement

No specific permits were required for the described field studies, and no specific permissions were required for these locations/activities. We confirm that these locations are not privately-owned or protected in any way and the field studies did not involve endangered or protected species.

### Insect Rearing and Sample Preparation

Stock colony of *Liposcelis bostrychophila* were developed from nymphs collected from a wheat warehouse in Chongqing, China and has been cultured in the Key Laboratory of Entomology and Pest Control Engineering, Southwest University, Chongqing since 1990. The insects were reared on a diet consisting of whole wheat flour, skim milk, and yeast powder (10∶1∶1) in an incubator at 27±1°C and 75–80% relative humidity with a scotoperiod of 24 h [15]. They were not exposed to blended gas or insecticides. To sample insects in different but uniform developmental stages, 100 plastic vials (1 cm in height by 2.4 cm in diameter with a nylon screen top) containing a small amount of diet were used to collect the uniform eggs. Fifty adults were aspirated into each vial. Forty-eight hours later, the adults were removed from the vials. According to the life history of *L. bostrychophila* at 27.5°C [51], developmental periods were used to discriminate the different stages. In brief, the developmental periods of 5, 11, 15, 19, 22, and 26 d were used for obtaining the eggs, first-, second-, third-, and fourth-stadium nymphs, and adults, respectively. Subsequently, the collected insects with the sample sizes of 1000, 1000, 800, 600, 500 and 500 for corresponding developmental stages above were used to extract the total RNA for transcriptome sequencing.

### Deltamethrin and Malathion Exposure

The test insects for insecticides exposure were the same as above, that is, susceptible line not exposed to blended gas and insecticides since 1990. Deltamethrin and malathion, both the most widely used grain protectants against stored products insects including psocids [7], were chosen to treat the insects according to the procedure described by Dou *et al*. (unpublished). The concentration LC_20_ was used as commonly adapted in pesticide exposure investigations of insects [22]. Deltamethrin (Sigma-Aldrich, St. Louis, MO, USA) and malathion (Sigma-Aldrich, St. Louis, MO, USA) were diluted in acetone at the concentration of 4.7 and 21.5 mg/L, respectively. For each insecticide solution, 300 µL was pipetted into glass Petri dishes (6 cm in diameter). Each Petri dish was rolled on its side until an even layer of insecticide dried on the inner surface. Newly emerged adults (3- to 5 days old) of *L. bostrychophila* were exposed to the deltamethrin and malathion layer, respectively, on the inner side of the Petri dish for 30 min. For each insecticide, 1,000 adults in total were exposed in 10 Petri dishes. Adults exposed to dishes coated with acetone served as a control. After 30 min exposure, treated insects were transferred into five fresh Petri dishes without insecticides or acetone and the insects were kept under the rearing conditions mentioned above. After 24 h of the exposure, surviving adults were collected and frozen in liquid nitrogen for RNA extraction. Approximately 800 adults for each treatment survived, and 500 adults were used for RNA extraction for each treatment. Three biological replicates were taken for each treatment.

### RNA Extraction and cDNA Library Preparation for Transcriptome Analysis

The RNA for each sample was isolated using the RNeasy Plus Micro Kit (Qiagen, Hilden, Germany). Genomic DNA was removed using a genomic DNA elimination column supplied with the kit. The total RNA of each sample was dissolved in 20 µL diethylpyrocarbonate (DEPC)-treated water and stored at −80°C. For each sample, RNA quantities were assessed at an absorbance ratio of OD_260/280_ (minimum ratio value of two) with a NanoVue spectrophotometer (GE Healthcare Bio-Science, Uppsala, Sweden). The integrity of RNA was confirmed by 1% agarose gel electrophoresis. Five micrograms of RNA from each developmental stage were mixed together to construct cDNA library. The integrity of the library was confirmed using both 1% agarose gel electrophoresis and the Agilent 2100 Bioanalyzer (Palo Alto, CA, USA) with a minimum integrity value of 7. RNA obtained from eggs, first-, second-, third-, and fourth-stadium nymphs, and adults was merged into one sample at equal ratios for transcriptome analysis.

Additionally, mRNA sequencing samples were prepared using the mRNA-seq sample preparation kit (Illumina, San Diego, CA). Following the Illumina manufacturer’s procedures, mRNA was purified from 10 µg of the pooled total RNA using polyT oligo-attached magnetic beads. Fragmentation buffer was added at 94°C for 5 min to disrupt the mRNA into short fragments (200–700 nucleotides). Reverse transcriptase and random primers were used to synthesize the first strand cDNA from the cleaved mRNA fragments. The second strand cDNA was synthesized using buffer, dNTPs, RNase H, and DNA polymerase I. These cDNA fragments were purified with the QIAquick PCR purification kit (QIAGEN, Hilden, Germany) and subjected to the end repair process that added an ‘A’ base to the 3′ end of the cDNA. Finally, the short fragments were connected with sequencing adapters. These ligation products were subjected to agarose gel electrophoresis and the suitable fragments were amplified by PCR to construct a cDNA library.

### Bioinformatics Analysis of Sequencing Results

The cDNA library was sequenced on the channels of an Illumina HiSeq™ 2000 instrument for 4 gigabases in-depth. The raw image data were transformed by base calling into sequence data (the raw reads). The clean reads obtained after filtering the raw reads were used for subsequent bioinformatics analysis. Transcriptome *de novo* assembly was performed with the short reads assembling programs Trinity, an efficient *de novo* transcriptome assembler, especially in the absence of a reference genome [52]. First, Trinity combined the reads with a certain overlap length to form longer fragments to generate sequence contigs. The individual reads were assigned to the respective contigs by paired-end mapping. Trinity was able to detect contigs from the same transcript and determine the distances between these contigs. Finally, Trinity connected these contigs into sequences that could not be extended on either end. Such sequences were defined as unigenes. After clustering with BLASTx, the unigenes were divided into two classes: clusters (similarity >70%) and singletons. The clusters were the transcripts produced by alternative splicing or homologous genes. All *de novo* assembled unigenes were used for BLASTx searches and annotations with a significant cut-off *E*-value of <10^−5^ against protein databases, such as Nr, Swiss-Prot, KEGG, and COG. The best matches were used to identify coding regions and to determine the sequence direction. If the alignment results of different databases conflicted with each other, we followed the priority order of Nr, Swiss-Prot, KEGG, and COG when determining the unigene sequence direction. Gene Ontology (GO, www.geneontology.org) functional annotation of unigenes was performed by Blast2go software [53]. COG and KEGG annotations were analyzed by the Blastall program against the Clusters of Orthologous Groups of proteins (COG, www.ncbi.nlm.gov/COG) and Kyoto Encyclopedia of Genes and Genomes (KEGG, www.genome.jp/kegg) databases. Besides, the sequencing depth (length of the unigenes divided by the total length of mapped reads) and coverage (length of the unigenes divided by the total length on unigene covered by reads) were analyzed. The database was submitted to the National Center for Biotechnology Information (NCBI) with Sequence Read Archive (SRA) submission number SRS390072.

### Analysis of Putative Pathways of Insecticide Detoxification and Target Proteins

Sequences encoding potential insecticide-detoxification enzymes (GSTs, CESs and P450s) were identified from the annotations in the Nr database with a cut-off *E*-value <10^−5^. The unigenes <400 bp in length were manually eliminated to permit accurate gene classification and localization in putative pathways; other sequences with ORFs <100 amino acids were also excluded. Those genes related to insecticide target proteins were also manually created using the above methods. The remaining GST, CES and P450 sequences were then searched by BLASTp against Nr. The unigenes found in the same BLAST results or with high homology to one another were eliminated as allelic variants or as different parts of the same gene. The putative pathways of these validated unigenes were identified using the KEGG database.

### Gene Expression Profile Sequencing

Samples for RNA isolation included *L. bostrychophila* adults exposed to deltamethrin, malathion, and acetone (control). Three DGE libraries of *L. bostrychophila* (control, deltamethrin-exposed, and malathion-exposed samples) were sequenced and the sequences have been deposited in GenBank with accession number PRJNA188391. Total RNA from these samples was extracted for digital gene expression (DGE) libraries that were prepared by the Illumina gene expression sample prep kit (Illumina, San Diego, CA). The mRNA was isolated from the total RNA using Oligo (dT) magnetic beads. The first and second strand cDNA was synthesized on the Poly(A) RNA-bound beads and subsequently digested by *Nla* III. The fragments cDNAs with 3′ ends were purified with magnetic beads and then Illumina adaptor 1 was added to 5′ ends of these cDNA fragments. After that, we used enzyme *Mme* I, which recognized the site of the junction of Illumina adaptor 1 and CATG, to digest these cDNAs fragments to produce 21 bp tags containing the adaptor 1. Subsequently, Illumina adaptor 2 was ligated to the tags at the 3′ ends, acquiring tags with different adapters at both ends and thus generating a tag library. After 15 cycles of linear PCR amplification, the required bands were purified by polyacrylamide gel electrophoresis. Then, the single-stranded molecules were sequenced via Illumina HiSeq™ 2000.

### Bioinformatics Analysis of Digital Gene Expression (DGE) Tags

To map the tags to the transcriptome database, the obtained raw sequence data were filtered by data processing steps to generate clean tags by a process that included the removal of adapter sequences, empty reads (reads with only adapter sequences but no tags), low-quality sequences (tags with unknown sequences ‘N’), and tags with a copy number of 1 (likely sequencing errors). A reference tag library containing all of the sequences of CATG plus 17 bases was created by searching the CATG sites in the transcriptome database. All clean tags were mapped to the reference library, permitting no more than a one-base mismatch. Clean tags, which were mapped to exactly one gene in the reference database, were designated as unambiguous tags for gene annotation. The number of unambiguous tags for each gene was calculated and normalized to TPM (number of transcripts per million tags) for the gene expression analysis [54].

### Screening and Analysis of Differentially Expressed Genes

We used the method described by Audic and Claverie [55] to identify differentially expressed genes (DEGs) between two different DGE libraries. The false discovery rate (FDR) method determines the P-value threshold for multiple testing by controlling the FDR value. The criteria of FDR ≤0.001 and the absolute value of log2ratio ≥1 were used to judge the significance of gene expression differences. For GO enrichment analysis of functional significance, we performed hypergeometric tests using the formula.
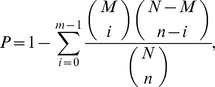
where N is the number of all genes with GO annotation, n is the number of DEGs in N, M is the number of all genes that are annotated to the certain GO terms, and m is the number of DEGs in M. A corrected *P*-value ≤0.05 was set as a threshold to identify the significant enrichment of GO terms in differential gene expression. The differentially expressed genes were also utilized in KEGG ontology (KO) enrichment analyses to further understand their biological functions. Pathway enrichment analysis could identify significantly enriched metabolic pathways or signal transduction pathways in DGEs compared with the genome background. Pathways with Q-value ≤0.05 were viewed as significantly enriched in DEGs.

### Validation of Gene Expression by qRT-PCR

qRT-PCR was performed to validate the mRNA sequencing data. Total RNA was extracted for the DGE library preparation as described above. One microgram of total RNA was reverse transcribed into single-stranded cDNA using the Primescript RT reagent kit (TaKaRa, Dalian, China). qRT-PCR was implemented using the SYBR premix Ex Taq kit (TaKaRa, Dalian, China), with the first strand cDNA serving as the template. The β-actin gene from *L. bostrychophila* was utilized as an internal control that was evaluated previously and applied as an appropriate reference for insecticide-induced gene expression profiling in the species [18]. The relative quantitative method (ΔΔC_T_) was used to calculate the fold change of target genes [56]. Three biological replicates were taken for each treatment. The primers employed in the qRT-PCR are listed in Table S3.

## Supporting Information

Table S1 **Kyoto Encyclopedia of Genes and Genomes database annotation of the** ***Liposcelis bostrychophila*** **transcriptome.**
(XLS)

Table S2 **The validated 10 genes (both up- or down-regulated) between the two comparison groups.**
(XLS)

Table S3 **The primers employed in the qRT-PCR.**
(XLS)
